# SARS-CoV-2 Testing and Complications Across 6 Waves of the COVID-19 Pandemic Among Individuals Recently Experiencing Homelessness in Ontario, Canada

**DOI:** 10.1001/jamanetworkopen.2023.12394

**Published:** 2023-05-08

**Authors:** Salimah Z. Shariff, Jennifer N. Reid, Andrew S. Boozary, Richard Booth

**Affiliations:** 1 ICES (formerly the Institute for Clinical Evaluative Sciences) Western, London, Ontario, Canada; 2 Arthur Labatt Family School of Nursing, Western University, London, Ontario, Canada; 3 Dalla Lana School of Public Health, University of Toronto, Toronto, Ontario, Canada

## Abstract

This cohort study compares the rates of SARS-CoV-2 testing and complications across 6 waves of the COVID-19 pandemic in Ontario, Canada, between individuals recently experiencing homelessness, low-income residents, and the general population.

## Introduction

Although numerous reports documented high rates of SARS-CoV-2 infections and complications among people experiencing homelessness early in the COVID-19 pandemic,^1^ an understanding of how this population fared as the pandemic progressed is limited. We compared the rates of SARS-CoV-2 testing and complications across 6 waves of the COVID-19 pandemic in Ontario, Canada, between individuals recently experiencing homelessness, low-income residents, and the general population.

## Methods

This cohort study follows the Strengthening the Reporting of Observational Studies in Epidemiology (STROBE) reporting guideline (eMethods in Supplement 1). The use of the data in this project is authorized under section 45 of Ontario’s Personal Health Information Protection Act (PHIPA) and does not require review by a research ethics board (REB). Projects that use data collected by ICES under section 45 of PHIPA, and use no other data, are exempt from REB review.

We conducted a matched retrospective cohort study using linked provincial SARS-CoV-2 laboratory testing, COVID-19 case and contact management, and health care administrative data. Study periods were stratified into 6 waves of the COVID-19 pandemic from March 1, 2020, to May 31, 2022. Adults with a recent experience of homelessness were matched on a 1:4 ratio on age and sex to the general population and residents living in the lowest-income neighborhoods of Ontario. We measured rates of reverse transcription–polymerase chain reaction testing, positive tests, percentage positivity, hospital admissions, hospitalizations requiring intensive care, and deaths associated with a SARS-CoV-2 infection in each wave. We used Poisson regression to calculate the rate ratio (RR) for all outcomes between individuals recently experiencing homelessness and their matched cohorts. All analyses were conducted at ICES using SAS, version 9.4 (SAS Institute). Two-sided *P* values less than .05 were considered significant; data analysis was completed between September 2022 and February 2023.

## Results

In total, 584 379 individuals were included in this study, of whom 65 524 (11.2%) had a recent experience of homelessness. The mean (SD) age of participants was 43.6 (16.5) years and 349 056 (59.7%) were male. Descriptively, all participant cohorts exhibited a similar pattern in rates of test positivity and complications of SARS-CoV-2 infection across the waves (Figure). In most situations, individuals recently experiencing homelessness experienced the highest magnitude in outcomes, followed by the low-income and general population cohorts. Of exception, percentage positivity was similar between individuals recently experiencing homelessness and low-income cohorts in the first 3 waves (RR ranging from 0.95-1.02) and lower among individuals recently experiencing homelessness thereafter (RR ranging from 0.55-0.82) (Table).

**Figure.**
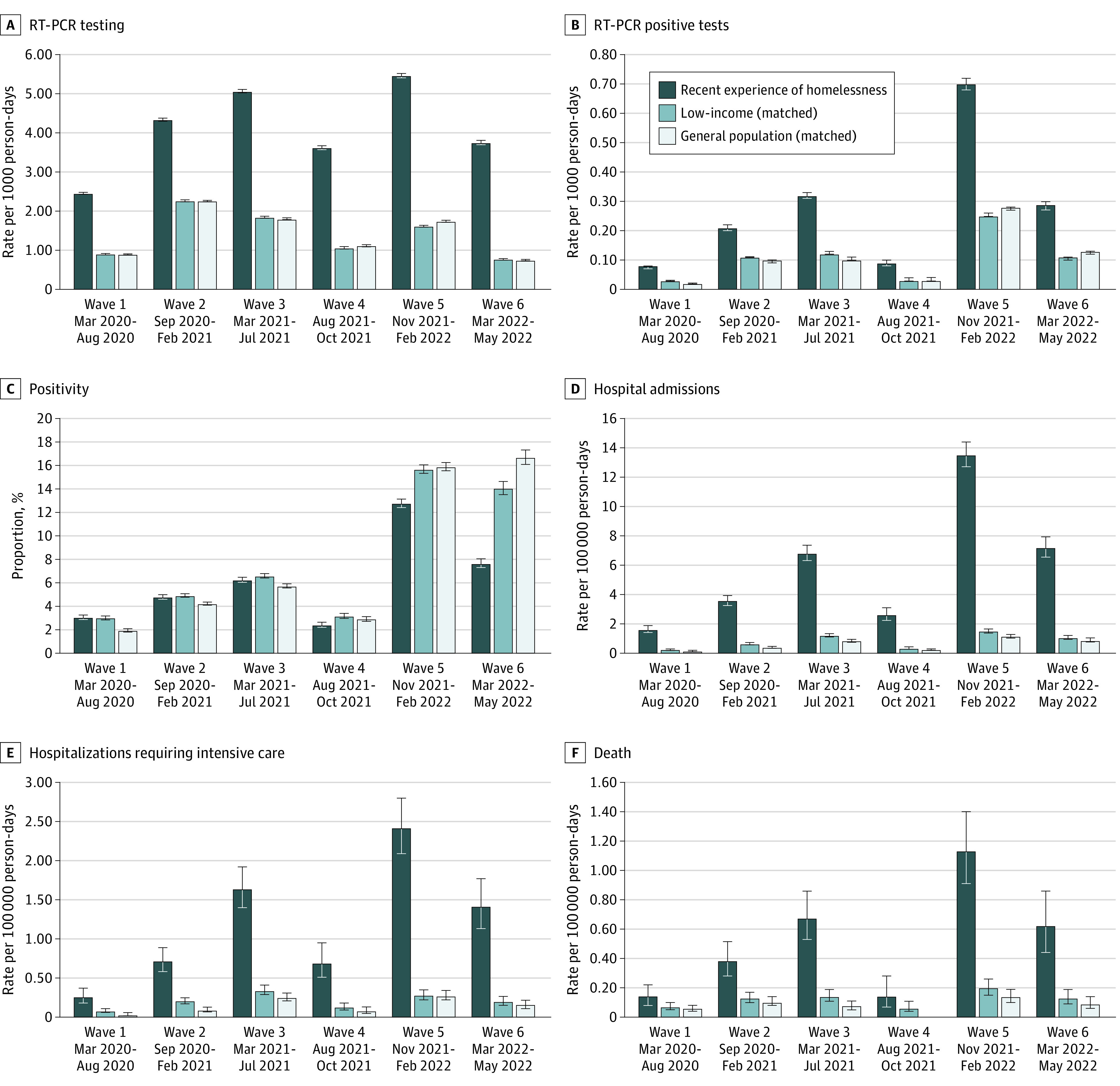
Rates of SARS-CoV-2 Testing, Test Positivity, and Complications Associated With SARS-CoV-2 Infections Across 6 Waves of the COVID-19 Pandemic From March 1, 2020, to May 31, 2022 RT-PCR indicates reverse transcription–polymerase chain reaction. Error bars indicate 95% CIs, calculated using the Rothman and Greenland method. Rates arising from 5 or fewer events are not presented in accordance with privacy requirements.

**Table.** Rate Ratios of SARS-CoV-2 Testing, Test Positivity, and Complications Associated With SARS-CoV-2 Infections Across 6 Waves of the COVID-19 Pandemic Between Individuals With a Recent Experience of Homelessness and Age-Matched and Sex-Matched Cohorts

Wave^b^	Rate ratio (95% CI)^a^	
	Individuals recently experiencing homelessness vs low-income	Individuals recently experiencing homelessness vs general population
**Outcome: RT-PCR testing**		
1	2.73 (2.69-2.77)	2.74 (2.7-2.78)
2	1.92 (1.90-1.94)	1.92 (1.90-1.94)
3	2.75 (2.71-2.78)	2.81 (2.78-2.84)
4	3.40 (3.34-3.47)	3.23 (3.17-3.29)
5	3.38 (3.33-3.42)	3.14 (3.10-3.18)
6	4.88 (4.78-4.98)	5.02 (4.92-5.12)
**Outcome: RT-PCR positive test**		
1	2.78 (2.55-3.02)	4.30 (3.91-4.73)
2	1.86 (1.78-1.96)	2.18 (2.07-2.29)
3	2.60 (2.49-2.73)	3.06 (2.92-3.21)
4	2.60 (2.32-2.91)	2.68 (2.39-3.00)
5	2.75 (2.66-2.85)	2.52 (2.43-2.61)
6	2.66 (2.50-2.84)	2.31 (2.17-2.45)
**Outcome: % positivity**		
1	1.02 (0.93-1.11)	1.57 (1.43-1.73)
2	0.97 (0.93-1.02)	1.13 (1.08-1.19)
3	0.95 (0.91-0.99)	1.09 (1.04-1.14)
4	0.76 (0.68-0.86)	0.83 (0.74-0.93)
5	0.82 (0.79-0.84)	0.80 (0.78-0.83)
6	0.55 (0.51-0.58)	0.46 (0.43-0.49)
**Outcome: hospital admissions**		
1	6.88 (5.45-8.69)	11.18 (8.49-14.71)
2	5.49 (4.73-6.37)	8.95 (7.52-10.66)
3	5.59 (4.96-6.30)	8.03 (7.02-9.17)
4	7.94 (6.01-10.50)	11.34 (8.27-15.55)
5	8.92 (7.97-9.99)	11.77 (10.39-13.32)
6	6.76 (5.75-7.95)	8.07 (6.80-9.57)
**Outcome: hospitalizations requiring intensive care**		
1	3.35 (2.06-5.43)	7.53 (4.10-13.81)
2	3.49 (2.59-4.69)	7.64 (5.28-11.05)
3	4.80 (3.80-6.05)	6.44 (5.00-8.30)
4	5.53 (3.40-8.99)	8.18 (4.73-14.15)
5	8.64 (6.64-11.24)	9.00 (6.90-11.75)
6	7.04 (4.86-10.18)	8.87 (5.96-13.22)
**Outcome: death**		
1	1.84 (1.02-3.32)	2.47 (1.33-4.61)
2	2.87 (1.94-4.24)	3.67 (2.42-5.54)
3	4.65 (3.24-6.67)	8.84 (5.70-13.71)
4	2.27 (0.95-5.41)	NR
5	5.62 (4.02-7.87)	7.98 (5.49-11.61)
6	4.82 (2.92-7.95)	7.13 (4.06-12.49)

Abbreviations: NR, not reportable; RT-PCR, reverse transcription–polymerase chain reaction.

^b^ Wave 1, March 1, 2020, to August 31, 2020; wave 2, September 1, 2020, to February 28, 2021; wave 3, March 1, 2021, to July 31, 2021; wave 4, August 1, 2021, to October 31, 2021; wave 5, November 1, 2021, to February 28, 2022; wave 6, March 1, 2022 to May 31, 2022.

^a^ Relative rates for all outcomes except percentage positive were calculated using generalized estimating equations with a Poisson distribution and log link, applying the person-time of follow-up per wave as an offset. Effect estimates for percentage positive were restricted to individuals who received 1 or more test in each wave and were estimated using a generalized linear model with a Poisson distribution and log link, applying the total number of RT-PCR tests per wave as an offset. Effect measures arising from 5 or fewer events are not presented in accordance with privacy requirements.

Both the magnitude and relative rates in complications of SARS-CoV-2 were most pronounced in the fifth wave. In this wave, hospital admissions among the cohort of individuals recently experiencing homelessness peaked at 13.5 (95% CI, 12.7-14.4) per 100 000 person-days compared with the low-income cohort (RR, 8.92 [95% CI, 7.97-9.99]) and general population cohort (RR, 11.77 [95% CI, 10.39-13.32]). Hospitalizations requiring intensive care occurred at a RR of 2.42 (95% CI, 2.09-2.80) per 100 000 person-days for the cohort of individuals recently experiencing homelessness compared with the low-income cohort (RR, 8.64 [95% CI, 6.64-11.24]) and general population cohort (RR, 9.00 [95% CI, 6.90-11.75]). Deaths occurred at an RR of 1.13 (95% CI, 0.91-1.40) per 100 000 person-days in the cohort of individuals recently experiencing homelessness compared with the low-income cohort (RR, 5.62 [95% CI, 4.02-7.87]) and general population cohort (RR, 7.98 [95% CI, 5.49-11.61]) (Table).

## Discussion

In this cohort study, hospitalizations and mortality associated with SARS-CoV-2 infection were found to be higher among individuals with a recent experience of homelessness across the first 6 waves of the COVID-19 pandemic compared with low-income residents and the general population of Ontario.

Prior to the emergence of the Omicron variant, absolute rates for all outcomes were greatest in wave 3, during which the Alpha variant was circulating. However, these rates were overshadowed by the excessive infections and complications observed in the first Omicron wave.^2^ These findings support ongoing strategies to mitigate SARS-CoV-2 exposure among people experiencing homelessness. Preparation for future pandemics should also consider longer-term supports for this population, who continue to be susceptible to adverse effects of infections more than 2 years into the pandemic.

This study was limited due to the administrative nature of our data. We were unable to determine the classification of homelessness among whom risks might differ. Further, with reductions in the availability and eligibility of SARS-CoV-2 testing along with increased reliance on rapid antigen tests for infection confirmation in our region starting December 2021,^3,4^ caution is warranted in the interpretation of surveillance and risk estimation efforts using health administrative data from this period and onwards.

## Notes

Supplement 1.
eMethods.

eReferences.

Supplement 2.
Data Sharing Statement
