# Supplementary material for: SARS-CoV-2 Testing and Complications Across 6 Waves of the COVID-19 Pandemic Among Individuals Recently Experiencing Homelessness in Ontario, Canada
Source: JAMA Netw Open. 2023 May 8;6(5):e2312394. doi: 10.1001/jamanetworkopen.2023.12394 (PMC10167569; doi:10.1001/jamanetworkopen.2023.12394)
Supplement: Supplement 1. — eMethods. eReferences. [file jamanetwopen-e2312394-s001.pdf]

Supplemental Online Content

Shariff SZ, Reid JN, Boozary AS, Booth R. SARS-CoV-2 testing and complications across 6 waves of the COVID-19 pandemic among individuals recently experiencing homelessness in Ontario, Canada. *JAMA Netw Open*. 2023;6(5):e2312394. doi:10.1001/jamanetworkopen.2023.12394

eMethods.

eReferences.

This supplemental material has been provided by the authors to give readers additional information about their work.

© 2023 Shariff SZ et al. *JAMA Network Open.*

eMethods

Setting: We conducted this study using healthcare administrative data from Ontario, Canada, which is Canada's largest province with an estimated population of 15 million residents in 2022.[1] With a single-payer healthcare system, residents of Ontario receive universal access to physician, hospital, and other healthcare services through the Ontario Health Insurance Plan, which are retained in prospectively collected health administrative databases. We conducted this study between March 1, 2020 and May 31, 2022, stratified by the first six waves of the COVID-19 pandemic in Ontario as determined by an assessment of the epidemiological curves of SARS-CoV-2 infections in the province:[2,3] wave 1: March 1, 2020 to August 31, 2020, wave 2: September 1, 2020 to February 28, 2021, wave 3: March 1, 2021 to July 31, 2021, wave 4: August 1, 2021 to October 31, 2021, wave 5: November 1, 2021 to February 28, 2022; wave 6: March 1, 2022 to May 31, 2022. Data used in this study were linked using unique encoded identifiers and analyzed at ICES (formerly the Institute for Clinical Evaluative Sciences). The use of data in this project is authorized under section 45 of Ontario's Personal Health Information Protection Act and does not require review by a Research Ethics Board.

Study participants: We applied a previously validated approach to assign individuals as recently experiencing homelessness[4,5] (population of interest) if they had one or more healthcare records with an indication of homelessness or unstable housing between January 1, 2019 (a period one year prior to the COVID-19 pandemic) and May 31, 2022 (throughout the follow-up period) in the Discharge Abstract, Same Day Surgery, National Ambulatory Reporting System, Ontario Mental Health Reporting System, Community Health Centre, or Case and Contact Management System databases. Individuals were identified if a healthcare record included an International Classification of Diseases, 10th Revision diagnosis code of Z59.0 (homelessness) or Z59.1 (inadequate housing), where residence type was documented as 'homeless' or 'shelter', or the patient was admitted from or discharged to 'Homeless

© 2023 Shariff SZ et al. *JAMA Network Open.*

(with or without shelter)'. The Canadian Institute for Health Information, the body responsible for the collection of national health administrative data, applies the following definition of homelessness "living on the streets or in places not intended for human habitation (e.g., sidewalks, parks, cars); staying in overnight shelters; and staying in temporary accommodations (e.g., motels, rooming houses, with friends/family, couch surfing, temporary housing for immigrants and refugees during settlement)".[6]

At the start of each wave, we retained recently homeless individuals who were alive, 18 years of age and over, and not living in a long-term care facility. These individuals were then matched (using greedy matching, without replacement) on a 1:4 ratio by age (exact) and sex at birth to (i) the general population and (ii) individuals living in the lowest income quintiles of Ontario.

Outcome definitions: Within the start and end dates of each wave, we calculated the rates of six outcomes for each participant cohort. We measured SARS-CoV-2 testing and positive tests using the COVID-19 Integrated Testing Dataset to identify all real time reverse transcription polymerase chain reaction (RT-PCR) tests based on the date of specimen collection. Among these tests, we measured percent positivity by dividing the number of positive tests over the total number of tests performed. We defined hospital admissions associated with SARS-CoV-2 infections as a hospital admission occurring seven (7) days prior to a positive test (to capture infections ascertained after hospital admission) to 30 days following a positive test, or a hospitalization with an International Classification of Diseases 10th revision diagnosis code of U07.1 (COVID-19). Among these hospitalizations, those requiring intensive care were retained for this outcome. Finally, using the Ontario Registered Persons and Case and Contact Management System databases, we identified deaths associated with a SARS-CoV-2 infections as those occurring 30 days following a positive test or seven (7) days prior to a positive test (for infection confirmation post-mortem), or where a death was recorded as directly associated with a SARS-CoV-2

© 2023 Shariff SZ et al. *JAMA Network Open.*

infection. Periods of time where participants were alive were used in calculating total person-days per wave (i.e., denominators used for rate calculations).

© 2023 Shariff SZ et al. *JAMA Network Open.*

eReferences

1. Government of Ontario Ministry of Finance. Ontario Demographic Quarterly: Highlights of first quarter; 2022. Accessed March 23, 2023. https://www.ontario.ca/page/ontario-demographic-quarterly-highlights-first-quarter

2. Public Health Ontario. *COVID-19 in Ontario: January 15, 2020 to June 14, 2022*; 2022. Accessed March 23, 2023. https://www.publichealthontario.ca/-/media/Documents/nCoV/epi/covid-19-daily-epi-summary-report.pdf?rev=5bda5a82fe2d4a7daedf292d5c298700&sc_lang=en

3. Public Health Ontario. Ontario COVID-19 Data Tool; 2022. Accessed March 23, 2023. https://www.publichealthontario.ca/en/data-and-analysis/infectious-disease/covid-19-data-surveillance/covid-19-data-tool?tab=trends

4. Richard L, Hwang SW, Forchuk C, et al. Validation study of health administrative data algorithms to identify individuals experiencing homelessness and estimate population prevalence of homelessness in Ontario, Canada. *BMJ Open*. 2019;9(10):e030221. doi:10.1136/bmjopen-2019-030221

5. Shariff SZ, Richard L, Hwang SW, et al. *Supplement to: Shariff SZ, Richard L, Hwang SW, et al. COVID-19 Vaccine Coverage and Factors Associated with Vaccine Uptake among 23 247 Adults with a Recent History o Homelessness in Ontario, Canada: A Population-Based Cohort Study.*; 2022. https://www.thelancet.com/cms/10.1016/S2468-2667(22)00037-8/attachment/e89d7fe8-1607-4392-9e5e-172cd1421ae4/mmc2.pdf

6. Canadian Institute for Health Information [CIHI]. *Canadian Coding Standards for Version 2018 ICD-10-CA and CCI.*; 2018. https://secure.cihi.ca/free_products/CodingStandards_v2018_EN.pdf

© 2023 Shariff SZ et al. *JAMA Network Open.*
