# Supplementary material for: SARS-CoV-2 Testing and Complications Across 6 Waves of the COVID-19 Pandemic Among Individuals Recently Experiencing Homelessness in Ontario, Canada
Source: JAMA Netw Open. 2023 May 8;6(5):e2312394. doi: 10.1001/jamanetworkopen.2023.12394 (PMC10167569; doi:10.1001/jamanetworkopen.2023.12394)
Supplement: Supplement 2. — Data Sharing Statement [file jamanetwopen-e2312394-s002.pdf]

Data Sharing Statement

Shariff. SARS-CoV-2 Testing and Complications Across 6 Waves of the COVID-19 Pandemic Among Individuals Recently Experiencing Homeless in Ontario, Canada. *JAMA Netw Open*. Published May 08, 2023. doi:10.1001/jamanetworkopen.2023.12394

Data

Data available: Yes
Data types: Other (please specify)
Additional Information: The datasets used in this study are held securely in coded form at ICES.
How to access data: While legal data sharing agreements between ICES and data providers (e.g., health care organizations and government) prohibit ICES from making the data set publicly available, access may be granted to those who meet pre-specified criteria for confidential access, available at https://www.ices.on.ca/DAS (email: das@ices.on.ca)
When available: With publication

Supporting Documents

Document types: Other (please specify)
Additional Information: The full data set creation plan and underlying analytic code are available from the authors upon request, understanding that the computer programs may rely upon coding templates or macros that are unique to ICES and are therefore either inaccessible or may require modification.
How to access documents: Corresponding author can be contacted for supporting documents.
When available: With publication

Additional Information

Who can access the data: Access may be granted to those who meet pre-specified criteria for confidential access, available at https://www.ices.on.ca/DAS (email: das@ices.on.ca).
Types of analyses: The full data set creation plan and underlying code.
Mechanisms of data availability: De-identified data may be granted to those who meet pre-specified criteria for confidential access, available at https://www.ices.on.ca/DAS.
